# Axial Spondyloarthritis: Reshape the Future—From the “2022 GISEA International Symposium”

**DOI:** 10.3390/jcm11247537

**Published:** 2022-12-19

**Authors:** Fausto Salaffi, Cesare Siragusano, Alessandra Alciati, Giulia Cassone, Salvatore D’Angelo, Serena Guiducci, Ennio Giulio Favalli, Fabrizio Conti, Elisa Gremese, Florenzo Iannone, Roberto Caporali, Marco Sebastiani, Gian Franco Ferraccioli, Giovanni Lapadula, Fabiola Atzeni

**Affiliations:** 1 Rheumatology Clinic, Ospedale Carlo Urbani, Università Politecnica delle Marche, 60035 Jesi, Italy; 2 Rheumatology Unit, Department of Experimental and Internal Medicine, University of Messina, 98125 Messina, Italy; 3 Department of Clinical Neurosciences, Hermanas Hospitalarias, Villa San Benedetto Menni Hospital, Como, and Humanitas Clinical and Research Centre, Rozzano, 20089 Milan, Italy; 4 Rheumatology Unit, Azienda Ospedaliera Policlinico di Modena, University of Modena and Reggio Emilia, 41125 Modena, Italy; 5 Rheumatology Institute of Lucania and Rheumatology Department of Lucania, San Carlo Hospital of Potenza, 85100 Potenza, Italy; 6 Department of Experimental and Clinical Medicine, University of Florence, 50134 Florence, Italy; 7 Division of Clinical Rheumatology, ASST Gaetano Pini-CTO Institute, 20122 Milan, Italy; 8 Department of Clinical Sciences and Community Health, Research Center for Adult and Pediatric Rheumatic Diseases, University of Milan, 20122 Milan, Italy; 9 Lupus Clinic, Dipartimento di Scienze Cliniche Internistiche, Anestesiologiche e Cardiovascolari, Sapienza University of Rome, 00161 Rome, Italy; 10 Rheumatology Unit, Fondazione Policlinico Universitario A. Gemelli IRCCS, 00168 Rome, Italy; 11 Rheumatology Unit, Department of Emergency Surgery and Organ Transplantations, University of Bari, 70121 Bari, Italy; 12 School of Medicine, Università Cattolica del Sacro Cuore, 00168 Rome, Italy

**Keywords:** axial spondyloarthritis, pain, pathogenesis, therapy

## Abstract

The term “axial spondyloarthritis” (axSpA) refers to a group of chronic rheumatic diseases that predominantly involve the axial skeleton and consist of ankylosing spondylitis, reactive arthritis, arthritis/spondylitis associated with psoriasis (PsA) and arthritis/spondylitis associated with inflammatory bowel diseases (IBD). Moreover, pain is an important and common symptom of axSpA. It may progress to chronic pain, a more complicated bio-psychosocial phenomena, leading to a significant worsening of quality of life. The development of the axSpA inflammatory process is grounded in the complex interaction between genetic (such as HLA B27), epigenetic, and environmental factors associated with a dysregulated immune response. Considering the pivotal contribution of IL-23 and IL-17 in axSpA inflammation, the inhibition of these cytokines has been evaluated as a potential therapeutic strategy. With this context, here we discuss the main pathogenetic mechanisms, therapeutic approaches and the role of pain in axSpA from the 2022 International GISEA/OEG Symposium.

## 1. Introduction

The term “axial spondyloarthritis” (axSpA) refers to a group of chronic rheumatic diseases that predominantly involve the axial skeleton and consists of ankylosing spondylitis, reactive arthritis, arthritis/spondylitis associated with psoriasis (PsA) and arthritis/spondylitis associated with inflammatory bowel diseases (IBD). AxSpA is classified into two major subtypes: the radiographic axSpA (rx-axSpA), also known as ankylosing spondylitis (AS), and non-radiographic axSpA (nr-axSpA), based on the presence or absence of inflammatory involvement of sacroiliac joints and/or spine on X-rays evaluation.

In both forms, active inflammation leads to pain, stiffness, and bone formation, and causes spinal mobility limitation and, thus, functional impairment. The peripheral joints and entheses, as well as skin bowel, and eyes, may also be involved [1,2]. Sacroiliitis is the axSpA distinctive clinical sign, and advanced imaging techniques, namely high-field magnetic resonance imaging (MRI), can obtain an early diagnosis and treatment.

## 2. Pathogenesis

The development of the AS inflammatory process is grounded in the complex interaction between genetic, epigenetic, and environmental factors and a dysregulated immune response, mainly involving the IL-23/IL-17 pathway [3,4,5]. The strongest association is with the human leukocyte antigen B27 (HLA-B27) of the major histocompatibility complex I (MHC-I), located on chromosome 6 [6]. The role of the MHC as a risk factor for several diseases is well established. The presence of HLA-B27 correlates with AS susceptibility and activity as it is positive in 80–90% of AS patients. The natural function of HLA-B27 is the binding and presentation of intracellular antigenic peptides to cytotoxic T lymphocytes: misfolding, the propensity to oligomerize and create complexes in the endoplasmic reticulum (ER) with the chaperone BiP (HSPA5/GRP78), is a biochemic peculiarity of HLA-B27. Together to other primary processes, misfolding has been hypothesized to explain the association between HLAB27 and AS. In fact, the growth of misfolded HLA-B27 may change ER function and induce a stress in ER causing a higher IL-23 production [7]. Moreover, alteration of the IL-23/IL-17 axis is the consequence of abnormal expression of HLA-B27 that could bind immunoglobulin-like receptor (KIR) of the Natural Killer (NK) cells, inducing an increased production of IL-17 [8]. Finally, alterations of the ERAP enzymes should provoke the formation of molecules defined as “arthritogenic peptide”. According to the ‘molecular mimicry’ theory a cross-reactive peptide, derived from an infecting pathogen, may stimulate T cells, which subsequently respond to an HLAB27 associated ‘self-peptide’, located in joints and entheses [9]. In the last few years, single nucleotide polymorphisms (SNPs) have been detected among the genes encoding the endoplasmic reticulum amino peptidase (ERAP) 1 and 2. These proteins are able to cut peptides in the endoplasmic reticulum with a variable length between eight and ten amino acids, which is optimal for binding to HLA-B27 [10]. The epistasis phenomenon links ERAP1 and HLA-B27, meaning that ERAP1 mutations involve only HLA-B27 positive patients [11].

Clinically, an overlap between the gut and joint inflammation has been well-documented. In about 60% of axSpA patients, there is evidence of subclinical gut inflammatory involvement, and 5–10% of them develop an inflammatory bowel disease (IBD). Several studies, including two genome-wide association studies (GWAS), have showed the presence of substantial genetic overlap between IBD and SpA. The composition of the extensive collection of bacteria colonizing the gastrointestinal tract and termed the ‘gut microbiota’, differs in healthy individuals or AS patients, along with an increase in IL-23 in the terminal ileum [12]. The perturbed gut microbiota affects the intestinal barrier on the epithelial and vascular side, as predicted by the gut-joint axis theory [13]. As a result of these processes, bacterial peptides and immunity cells move towards the interstitium; subsequently, they move into the circulation, inducing an abnormal systemic inflammatory response [14]. The bacterial dysbiosis theory suggests that the expression of similar receptors between the synovial membrane and the gut epithelium may lead to joint inflammation, due to a decrease in HLA-B27-mediated clearance of intracellular bacteria.

Pathogenesis of inflammation and bone formation in the entheses are complex, and probably involve mechanical stress, as suggested by studies showing that the anterior longitudinal ligament is the most affected in patients with AS, bearing a higher load [15].

Considering the pivotal role of IL-23 in AS, it is expected that the suppression of this cytokine is an effective therapeutic approach. However, Ustekinumab failed to demonstrate a significant efficacy on pain and inflammation in radiographic axSpA, in a phase III clinical trial [16]. Accordingly, Risankizumab, a monoclonal antibody binding p19 to IL-23, did not show significant differences when compared to placebo in a phase II study on axSpA [17].

The Ineffectiveness of monoclonal antibodies against anti-IL-23 in AS patients prompted a revaluation of the mechanisms of action supposed until then, based on in vitro data and preclinical models. It was previously demonstrated that Th-17 cells may arise from both IL-23-dependent and -independent pathways, and that different populations of T cells (γδ T cells, type 3 innate lymphoid cells and mucosal-associated invariant T cells) produce IL-17 [18]. These results allow us to hypothesize different mechanisms to explain the poor efficacy of the IL-23 blockade in axSpA.

The interaction of mesenchymal and immune cells fosters the inflammation of synovia in axSpA [19]. The possibility that the decoupling of IL-23 and IL-17 can be tissue specific is supported by in vitro studies showing that skin mesenchymal cells trigger the release of IL-17 through an IL-23-dependent pathway, while synovial mesenchymal cells trigger the release of IL-17 by activated IL-23-independent T cells [20,21]. The effectiveness of IL-23 blockades for the treatment of peripheral and not for axial involvement could be due to the low number of monocytes and dendritic cells in axial tissues, but a careful definition of these cellular population has not yet been evaluated. Another possible explanation may be that IL-17-producing cells reach the sacro-iliac level from other sites, already activated by IL-23.

The presence of an axis between gut and joint may explain the link between dysbiosis and inflammatory diseases of the spine, but not the lack of efficacy of anti-IL-23 in axSpA, since this treatment should involve cellular lines in both bowel and spine.

As in rheumatoid arthritis [22], IL-23 appeared to promote the first phase of AS, but not necessarily maintain the ongoing inflammation. Although the IL-23 axis alters the glycosylation of self-reactive IgG antibodies, making them pathogenic, this is unlikely to be the mechanism through which IL-23 promotes the onset of AS, probably triggered by alternative pathways. Another explanation for the early involvement of IL-23 in AS pathogenesis is that the IL-23/IL-23R complex may act on different effectors and transducers that are not suppressed by anti-IL-23 antibodies [23].

The serum levels of IL-17A and IL-17F are significantly higher in axSpA patients than in healthy subjects [24,25] and they are significantly related to disease activity [26,27].

Some studies have demonstrated that the terminal ileum of AS patients is a significant source of IL-23 but not IL-17 [12], and that IL-23p19 (the unique subunit of the active IL-23 cytokine) is overexpressed in the inflamed gut tissues. Accordingly, IL-17F is more frequently observed and more expressed in the synovial tissue of patients with PsA than with osteoarthritis [28,29].

IL-17A has a synergic effect in combination with other cytokines, leading to a higher pro-inflammatory response, despite these effects have been poorly explored in axSpA patients.

Although the role of IL-17F in the pathogenesis of axSpA has to be furtherly explored, it is established that the inflammation process derives by a synergic effect of IL-17A and IL-17F [30] and that the blockade of both IL-17A and IL-17F decreases the inflammatory response better than the blockade of only IL-17A [30].

The mechanism that allows the synergistic effects of IL-17A and IL-17F remains unclear. Some Authors has suggested that IL-17A might stabilize mRNA transcripts, and increase gene expression and protein production [31]. Moreover, phospholipase D enzymes might up-regulate the cytokine secretion. It is worth noting that despite the absence of evidence concerning IL-17F, L-8 mRNA and other mRNA transcripts (including ACT1, MIP2 and CSF2) may have a role in the synergy between IL-17A and TNF [32].

Many of the immunity cell populations capable of producing IL-17, (IL-17+ CD8+ T cells, tissue-resident memory (TRM) T cells, MAIT cells, Invariant NKT cells, γδ T cells) may secrete IL-17 in patients with axSpA [33,34,35].

## 3. Treatment

The recommendations of the Assessment of Spondyloarthritis International Society/European League Against Rheumatism (ASAS/EULAR) and those of the American College of Rheumatology/Spondylitis Association of America/Spondyloarthritis Research and Treatment Network (ACR/SAA/SPARTAN) [36] suggest therapy with non-steroidal anti-inflammatory drugs (NSAIDs) and physical treatment as the first-line therapy of pain and stiffness. Since conventional disease-modifying anti-rheumatic drugs (DMARDs) such as methotrexate, sulfasalazine, and leflunomide are ineffective in treating the axial manifestations of axSpA, their role is limited to the treatment of peripheral clinical manifestations of axSpA.

The biological DMARDs (mainly TNF and IL-17 inhibitors) are strongly recommended for patients who do not respond to NSAIDs or when NSAIDs are contraindicated. The compound choice should be based on the interaction between safety and comorbidity, taking into account the presence of extra-articular manifestations and patient preference, as there is no indication that any available drug is more effective than the others. Clinical trials are the gold standard to assess the new biological drug efficacy and safety. However, as the clinical trials undergo rigorously standardized conditions and patient inclusion criteria, their results may not be indicative of their prescription and use in real-world.

### 3.1. Anti-IL17

Until a few years ago, the TNF inhibitors were the only biological agents licensed to treat AS and nr-axSpA. The introduction of the first two IL-17 inhibitors (secukinumab [SEC] and ixekizumab [IXE]) has increased the opportunities for the treatment of patients who do not respond to TNF inhibition or experiencing a secondary failure. Moreover, three other compounds (bimekizumab, brodalumab and netakimab) are now in different stages of clinical development and approval [37].

#### 3.1.1. Secukinumab

A phase II [38] and five phase III trials (MEASURE 1 [39], MEASURE 2 [40], MEASURE 2-J [41], MEASURE 3 [42] and MEASURE 4 [43] and their extensions tested the efficacy and safety of SEC, a fully human antibody against IL-17A that inhibits the interaction between IL-17 and its receptors [44]. These studies demonstrated rapid and sustained efficacy in clinical and radiologic endpoints in AS, without evidence of a reduced efficacy and a favorable and significant safety profile over a 5-year period.

PREVENT is the first phase III trial of SEC in patients with active nr-axSpA [45] that demonstrates an improvement of signs and symptoms during the 52-week study period without significant safety findings in patients treated with SEC. The two studies met both of their primary outcomes. Specifically, SEC 150 mg with loading doses than in those receiving PBO demonstrated a higher ASAS40 response rate at 16-week (41.5% vs. 29.2%; *p* = 0.0197), and a higher 52-week ASAS40 response rate also in patients receiving SEC 150 mg without loading doses (39.8% vs. 19.9%, respectively; *p* < 0.0021) [45].

In the multicenter, prospective, observational Spondyloarthritis Roman Group (STRONG) study, SEC was able to improve all of the evaluated clinical features and patient-reported outcomes after six and twelve months of therapy, in particular among male AS patients, in the absence of significant side effects [46].

Another observation study, recruiting 1860 axSpA patients from 13 European registries of the European Spondyloarthritis Research Collaboration Network [47], showed that the SEC retention rates was 82% after 6 months and 72% after 12 months of treatment thus similar to those reported in studies evaluating TNF inhibitors. The response rates observed in observational studies were lower than those obtained in randomized clinical trials, but they were usually better among biological drug-naïve patients. Recently, a systematic review and meta-analysis of real-world data on biological therapies for the treatment of AS showed a one-year drug survival rate of SEC of 0.77 (95% confidence interval 0.64–0.90) [48].

#### 3.1.2. Ixekizumab

Two phase III RCTs (COAST-V and COAST-W) showed significant ASAS40 responses after the administration every two or every four weeks of IXE, an IgG4 monoclonal antibody binding the homodimer IL-17A and the heterodimer IL-17A/F, in the treatment of radiographic axSpA [49,50].

#### 3.1.3. Other IL-17 Inhibitors

Brodalumab is nowadays approved for the treatment of psoriasis [51]. It is an IL-17A receptor antagonist also inhibiting IL-17F, the IL-17A/F heterodimer and IL-17E. Unfortunately, because of concerns about suicidal behavior [52], despite the absence of a demonstrated causal relationship, a phase III trial evaluating the efficacy of Brodalumab in patients with PsA was suspended. Similarly, a placebo-controlled phase II trial evaluating Brodalumab in axSpA patients (ClinicalTrials.gov ID NCT02429882) was interrupted and withdrawn in 2015. During the 2019 EULAR meeting, the results of a phase III trial on brodalumab in AS and nr-axSpA patients (ClinicalTrials.gov ID NCT02985983) performed in Japan were reported [53]. The results of the study showed a significantly higher response rate at week-16 ASAS40 in the brodalumab group (35/80, 43.8%, *p* = 0.018) when compared to placebo (19/79, 24.1%). These results suggested a possible future role for brodalumab as a therapeutic alternative for axSpA patients.

Bimekizumab is an antagonist of both IL-17A and IL-17F. A phase IIb trial recruiting AS patients [54] demonstrated that patients treated bimekizumab every four weeks achieved a significantly better week-12 ASAS40 response than patients receiving placebo (response rate for bimekizumab 16 mg: 29.5%, bimekizumab 64 mg: 42.6%, bimekizumab 160 mg: 46.7%, while the response rate in PBO group was vs. 13.3%; *p* < 0.05) [55]. Phase II trials involving AS patients (ClinicalTrials.gov ID NCT03355573 and NCT03215277) and phase III trials including patients with AS (ClinicalTrials.gov ID NCT03928743), nr-axSpA (ClinicalTrials.gov ID: NCT03928704), or both AS and nr-axSpA (ClinicalTrials.gov ID NCT04436640) are currently ongoing.

Finally, Netakimab is a recombinant humanized IgG1 monoclonal antibody against IL-17 with a modified Fc fragment and CDR regions. A phase III trial, placebo-controlled (ClinicalTrials.gov ID NCT03447704) is currently ongoing to evaluate safety and efficacy of a dose of 120 mg over one year in 228 patients with active AS [55].

### 3.2. IL-23 Inhibition

Despite post-hoc analyses of trials on PsA [56,57,58] suggested that Guselkumab and Ustekinumab can improve back pain symptoms potentially induced by axial inflammation; this is probably due to generic and non-specific effects [59]. Globally, the experimental data suggest that IL-23 is not a relevant driver of the physiopathology and clinical features of active axSpA [60].

#### 3.2.1. Ustekinumab

In a prospective, open-label, proof-of-concept clinical trial, Ustekinumab treatment reduced active AS signs and symptoms without significant adverse effects [61], but three placebo-controlled trials fail to confirm its efficacy [16,62]. The first two studies evaluated Ustekinumab treatment of radiographic axial SpA by including patients naïve to TNF inhibitors and those that failed to respond or were intolerant to TNF inhibitors, respectively. A third study included patients with nr-axSpA. For all three studies, all of the Ustekinumab dose groups showed clinically significant improvement over placebo on key efficacy outcomes. The percentage of patients that experienced adverse events in the Ustekinumab groups was consistent with that one observed in previous studies [16].

#### 3.2.2. Guselkumab

A study on subcutaneous Guselkumab in patients naïve to biologic drugs with active PsA and axial involvement (STAR) is ongoing (ClinicalTrials.gov Identifier: NCT04929210) [62].

#### 3.2.3. Risankizumab

Risankizumab is a humanized IgG1 monoclonal antibody binding the p19 subunit of IL-23 [62]. Efficacy and safety of Risankizumab in AS patients were evaluated in a randomized, double-blind, placebo-controlled, phase II study. A treatment with Risankizumab failed to achieve the primary outcome of ASAS40 response at week 12 (obtained by 25.5%, 20.5%, and 15% of patients in the Risankizumab 18 mg, 90 mg, and 180 mg groups, respectively, compared to 17.5% in the placebo group). Adverse events were similar comparing Risankizumab and placebo groups [17]. These results do not make recommended the use of Risankizumab in axSpA.

#### 3.2.4. Tildrakizumab

Tildrakizumab is a IgG1 subclass monoclonal antibody that binds to human IL-23. The preliminary results of a randomized, placebo-controlled, double-blind, phase IIa study on the treatment of active AS or non-radiographic axSpA (ClinicalTrials.gov Identifier: NCT02980705) showed the ineffectiveness of Tildrakizumab, according to previous studies on axSpA evaluating treatments with a similar mechanism of action [62].

### 3.3. Janus Kinases Inhibitors

In the last few years, several treatments have been investigated and approved for treating patients with axSpA with different and controversial results. Today, the new class of drugs called Janus kinases inhibitors (JAKi) have been approved for treating patients with rheumatoid arthritis, PsA and recently for axSpA. Clinical trials in axSpA showed that JAKi are effective and safe for treating these patients with a particular effect on pain.

## 4. The Impact of Pain in SpA Management

As in most rheumatic diseases, pain is an important and common symptom of axSpA, and may include periods of both fluctuating and more persistent pain [63,64]. Additionally, it may progress to chronic pain, a more complicated bio-psychosocial phenomena, comprised of chronic widespread pain (CWP) and chronic localized pain [65]. Chronic pain is now recognized as a separate disease in and of itself, in addition to being the primary symptom of rheumatic disorders. Central sensitization (CS) ensures the continuation of chronic pain. CS is an atypical mechanism of pain control involving the central nervous system [66]. CS synaptic plasticity is a condition characterized by an increase in neuronal responsiveness in central pain pathways in response to painful stimuli. This is a significant non-nociceptive pain mechanism that results from altered central nervous system pain processing and may occur in the absence of peripheral injury or inflammation [67]. According to current knowledge, CS is caused by peripheral and central nervous system neuroinflammation. Consequently, cytokines and chemokines are released into the spinal cord and brain. Cytokines and chemokines in the central nervous system are important neuromodulators in the development of hyperalgesia and allodynia [68]. Additionally, neuroimaging analysis revealed significant cortical thinning in a number of brain locations (e.g., primary somatosensory cortex, insula, anterior middle cingulate cortex), but increased gray matter volume in the putamen and thalamus [69]. These data strongly suggest that the neuropathic component of AS may be related with central processes. Psychosocial factors contributing to CS and somatosensory changes are depression, anxiety, stress, and cognitive factors, including catastrophizing and maladaptive illness perception. Additionally, it has been shown that inflammatory and immune system processes in both the peripheral and central nervous systems are unquestionably involved in neuropathic pain (NP). Koca et al. [70] discovered a correlation between CS and NP scores and disease activity ratings, and they recommended including central pain management techniques into the diagnosis and follow-up of AS. Some researchers have identified widespread pain in axSpA patients [71]. The occurrence of NP in various rheumatic diseases was explored using the Pain Disability Questionnaire (PDQ) in a large study of 7.054 patients enrolled in DANBIO (Denmark’s national registry of biological treatments) [72]. NP characteristics were seen in 20% of individuals with rheumatoid arthritis (RA). This trait was seen in 28% and 21% of patients with PsA and axSpA, respectively. All patient-reported outcomes were greater in this subgroup of patients, reporting increased pain, exhaustion, and disability, as well as worse global health. In comparison, no variations in C-reactive protein (CRP), serology, or current biological therapy were observed. The prevalence of a neuropathic component in SpA has yet to be extensively studied and described in detail, with special emphasis on the differentiation from nociplastic pain.

The current definition of NP, according to the International Association for the Study of Pain (IASP), is “pain induced by a lesion or disease of the somatosensory nervous system” [73]. Choi et al. found NP in 14.2% of 105 AS patients [74] using the Pain-DETECT questionnaire, while Gok et al. found it in 33.5% of AS patients using the same tool [75]. We ruled out NP and identified CS using the Central Sensitization Inventory (CSI) questionnaire score. The CSI was designed and validated to detect chronic (nociplastic) pain in individuals suffering from chronic pain (Table 1).

**Figure 1:**
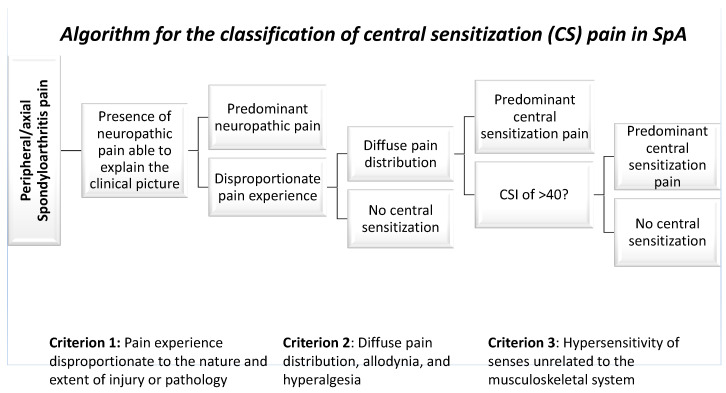
The classification of central sensitization (CS) pain requires two major steps: the exclusion of neuropathic pain and the differential classification of nociceptive versus central sensitization pain. The algorithm for CS pain categorization in SpA is described here.

The CSI has previously been used in osteoarthritis, rheumatoid arthritis, SpA, Inflammatory Bowel Disease (IBD) and fibromyalgia [76,77,78,79]. To differentiate predominant nociceptive and CS pain, clinicians are advised to use the algorithm shown in the Figure 1, guiding them through the screening of three major classification criteria. Although their pathophysiological causes are distinct, nociplastic pain and NP have similar clinical features and are difficult to distinguish using simply questionnaires.

To further understand the feature of CS-related pain in SpA, particularly the association with CSI scores and disease activity evaluations, studies using methodologies such as quantitative sensory testing (QST), including pressure pain thresholds and conditioned pain modulation testing, are required.

In individuals with fibromyalgia (FM), Macfarlane et al. [80] discovered a strong association between the two components (Symptom Severity Scale and Widespread Pain Index). FM is a common condition that has been linked to CS [79,81]. FM is characterized by widespread pain, fatigue, and sleep disturbances, similar to SpA. Patients with FM can be identified using the 2016 revision of the American College of Rheumatology (ACR) criteria [82], which include the presence of chronic pain for at least 3 months, a widespread pain index (WPI) of 7 and a symptom severity scale (SSS) score of 5 or a WPI of 4–6 and an SSS score of 9 or a WPI of 4–6 and an SSS score of 9 [81]. According to Kieskamp et al., 45% scored 40 or above, suggesting a significant likelihood of CS [83]. As a result, people who have had axSpA for a long time seem to have a higher risk of developing CS. This is in line with a previous study of 200 axSpA patients with a mean symptom duration of 5.9 years and a mean Ankylosing Spondylitis Disease Activity Score (ASDAS)-CRP of 3.2, in which a disproportionate number of patients (24%) met the ACR criteria for FM [84]. The overall prevalence of FM was 14.9% in the axSpA population, with women having a considerably higher prevalence (*p* < 0.0001); the estimated prevalence in AS was 12.7%, and in axial PsA, it was was 17.2% [81,85].

In conclusion, there are two fundamental processes in the categorization of CS pain: the exclusion of NP and the differential classification of nociceptive vs. CS pain. Within the classification algorithm (Figure 1), the criteria should be examined together [86]. The existence of NP or vice versa does not rule out the possibility of CS. In patients complaining of persistent pain, therapy of centrally sensitized diseases should be priority, particularly in those whose condition has been managed with proper treatment. CS is a rather prevalent disorder, and pain-coping strategies, in addition to pharmacological therapies, may help individuals with SpA reduce pain and improve their quality of life. It’s critical to increase awareness about the critical role that CS pain plays in these individuals’ lives.

## Figures and Tables

**Table 1:** Central sensitization inventory: part A.

1	I feel tired and unrefreshed when I wake from sleeping.
2	My muscles feel stiff and achy.
3	I have anxiety attacks.
4	I grind or clench my teeth.
5	I have problems with diarrhea and/or constipation.
6	I need help in performing my daily activities.
7	I am sensitive to bright lights.
8	I get tired very easily when I am physically active.
9	I feel pain all over my body.
10	I have headaches.
11	I feel discomfort in my bladder and/or burning when I urinate.
12	I do not sleep well.
13	I have difficulty concentrating.
14	I have skin problems such as dryness, itchiness, or rashes.
15	Stress makes my physical symptoms get worse.
16	I feel sad or depressed.
17	I have low energy.
18	I have muscle tension in my neck and shoulders.
19	I have pain in my jaw.
20	Certain smells, such as perfumes, make me feel dizzy and nauseated.
21	I have to urinate frequently.
22	My legs feel uncomfortable and restless when I am trying to go to sleep at night.
23	I have difficulty remembering things.
24	I suffered trauma as a child.
25	I have pain in my pelvic area.

Part A of the Central Sensitization Inventory (CSI) assesses 25 health-related symptoms common to CSSs. Responses are recorded about the frequency of each symptom, with a Likert scale from 0 (never) to 4 (always), resulting in a total possible score of 100. Higher overall scores indicate more CS symptoms. A cut-off point of 40 out of 100 is suggestive for the presence of CSS. CSS severity categories are classified as subclinical (0–29), mild (30–39), moderate (40–49), severe (50–59), and extreme (60–100).
